# Solid, Gel, and Solvent of Intensive Care Unit Leadership

**DOI:** 10.7759/cureus.13479

**Published:** 2021-02-22

**Authors:** Iqbal Ratnani, Sahar Fatima, Faisal Masud

**Affiliations:** 1 Critical Care Medicine, Houston Methodist Hospital, Houston, USA

**Keywords:** leadership, ethics, framework, guidelines, medical education, creativity, connectivity, adaptability, transparency, followership

## Abstract

The first leadership emerged simultaneously at the time when humans started forming groups to fight against a common threat or to attain means of survival for food, shelter, and safety. Authors have tried to define, understand, and apply the context of leadership in different social, cultural, political, organizational, and religious setups. This article will describe three aspects of leadership to encompass comprehensive traits of strong leadership in a particular reference to any multidisciplinary intensive care units (ICUs) in a tertiary care hospital.

## Introduction and background

The history of leadership was never written since the origin of mankind. Humanity has witnessed all sorts of leaders from authoritarian to servant-ship in forms of tribal leaders, emperors, invaders, dynasties, democratically elected, and other various types. The authors of this article have coined the term SolGelSol which stands for three words, i.e., solid, gel, and solvent. A leader in his solid role is not only expected to provide dynamic guidelines for daily work but also create a boundary of ethics for his teams. A leader is supposed to provide context to team members of different intensive care units (ICUs), with the relevant healthcare system and society in general. A leader adapts like a gel to incoming challenges, rolls his team towards positivity, and away from negativity, adversity, hostility, and uncertainty. In the gel form, tends to change the shape, size, and density of the team in a given situation. As a solvent, integrates with all his team members. Becomes a catalyst for the team morale and a powerhouse of enthusiasm between subgroups of his organization. Good leadership is vital for ICUs in any healthcare system. All essential benchmarks like quality, safety, teaching, work environment revolve around leadership and have long-reaching impacts on patients and their families' wellbeing.

## Review

Value vs action

**Figure 1:**
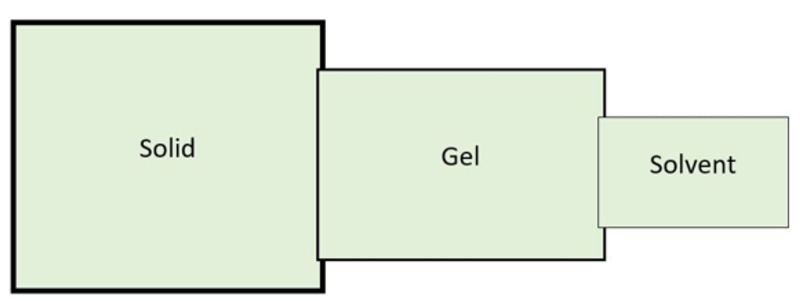
The fluidity of SolGelSol

**Figure 2:**
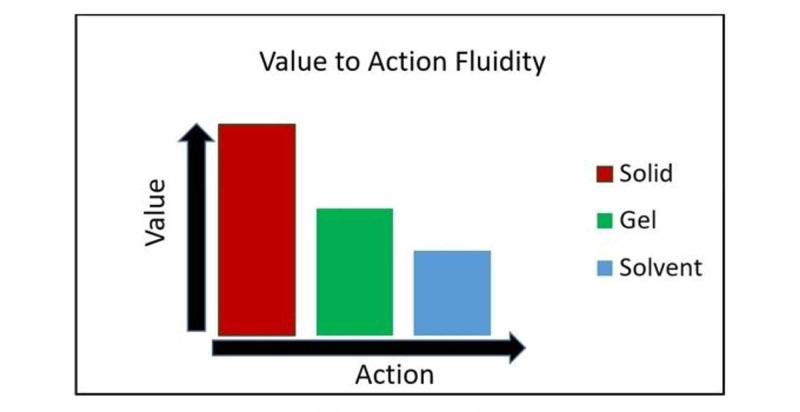
Weightage of leader's value to action

The concept of leadership has evolved in the last century [1]. The build of these three fundamentals of leadership runs on the pattern of the fluidity concept of legendary American architect Frank Lloyd Wright [2]. All of the three fundamentals of leadership are equally important and related. Responsibilities of a group leader range from providing ethical guidance to self-involvement at ground zero. As a leader, he continuously balances the components of values and actions. He constantly juggles between providing frameworks, guidelines, ethics, context, adapting to challenges, and having direct involvement. He preserves his credibility by leading an example as highlighted by Baldoni [3]. If needed he will not shy away from doing simple but vital actions like replacing sanitizers in ICUs, guiding families in hallways or helping his staff to run "code," or assisting students in procedures. The concept has been presented in diagrammatic forms. In Figure 1, the fluidity of SolGelSol has been emphasized with the sizes and margins of the boxes, and in Figure 2 with bar densities on x and y axes of values and action, respectively.

Components of Solid Aspect

Establishing boundaries of ethics:* *No organization of any nature or any magnitude can survive without its core values and boundaries of ethics [4]. And, this becomes even more critical in an ICU setting. When a leader speaks of ethics, it encompasses mission, vision, governance, and leadership itself [5]. He defines the boundaries of an ethical framework for everyday work and duties of the team members, quality of the organization, due processes, fairness during conflicts, and use of resources [6]. Intense practice of ethics pays off in the long run. He will not shy away from "correction," as we knew from Netflix’s Reed Hastings’ 2011 famous words, “I messed up. I owe you an explanation.” This was after backlash from customers on subscription hikes [7]. On the contrary, Blockbuster went on bankruptcy, which thrives on charging late fees from its customers, and refused to revert it despite clear signs [8]. These ethics and values can be formally written as the ICU’s policy or may just be practiced informally and frequently mentioned in communications. In fact, medical ethics has become a formalized study. The study of medical ethics and ethical leadership are different from each other. Medical ethics applies to the practice of clinical medicine and research. It includes the four principles of respect for autonomy, beneficence, non-maleficence, and justice. Ethical leadership deals with naming values pertinent to the organization. It may be of utmost importance to name value as “We Can’t Live into Values That We Can’t Name” [9].

Providing context*:* Team members, which include ICU physicians, nurses, and others, many times may be lost in putting context to their higher undefined role inside the organization as well in relating to the outside world. In addition, they may not be able to co-relate their positions in terms of the broader objectives of the organization. A leader establishes context [10]. It's all about keeping the overall health of an organization [11]. It's about providing context to five essentials, i.e., environment, strategy, culture, organizational complexity, and stakeholders’ expectations [12]. A leader determines context based on five "Ws" of "who, what, when, and where you lead determines how you lead" [13]. Mnemonic has been described to encompass the full contextual values a leader should provide from a personal level to the outside environment. Mnemonic is SOTOA, which stands for Self-Context, One-to-One Context, Team Context, Organizational Context, and Alliance Context [13].

Laying out frameworks and guidelines*: *Micromanaging can kill an organization. Though a leader gets involved with his team at ground level (as discussed below in the solvent section), but he tends to provide a broader framework and general guidelines. Hence, the integrity of an organization stays intact. He creates a leader in every person in the team. "Leaders don't create followers; they create more leaders" [14]. It can be via many strategies like defining the team's boundaries of responsibility, delegation, avoiding abdication, periodic debriefing, and updates, managing outcomes but not processes, coaching, encouraging peer-to-peer empowerment, setting specific and measurable goals, and respecting the chain of commands [15].

His framework provides a bigger picture with the prioritization of truly important targets. His framework and guidelines empower, nurture, inspire, and symbiotically thrive on the followers' strength. Instead of making a cult, he builds a community out of his followers [16].

Components of Gel Aspect

Adaptability: Charles Darwin said: “The most important factor in survival is neither intelligence nor strength but adaptability.” Healthcare challenges are moving targets. The leader models adaptability and encourages his ICU and the team to adjust accordingly. This ongoing process requires constant vigilance. Being a leader, he has to be quick and proactive to forecast incoming challenges, anticipate dynamics of adaptability, and vision constant growth in a continually changing environment. In the modern world, it calls for embracing technology, like the electronic medical record (EMR) at every desk [17,18]. Stephen Zaccaro is a well-known organizational psychologist. He developed a framework of three flexibilities of adaptability: cognitive, emotional, and dispositional. The last one refers to have an ability to be simultaneously optimistic as well as realistic [19]. For a leader, a compromise is not a weakness but a virtue [20]. A good leader strives to be a careful mix of both persistence and flexibility, at least if not a full-fledged chameleon [21].

Creativity: Gel can be molded into many new forms. A leader has the vision and creativity to continuously pursue newer concepts to grow and expand (or retract) his organization. It’s all about innovations. It’s about putting research into the organization and bringing it to practice [22]. The creativity of leadership knows to seek opportunity through the tension between order and chaos, and commerce and self-expression [23]. A creative leader rattles the cages, listens to his and his teams’ intuitions and convictions. With his gel property, he let the whole organization think out of the box besides what’s expected of them [24]. Although creativity tends to be a personality trait, it requires a deliberate effort [25]. Creativity can be harness by anyone and everywhere [26]. One of the biggest challenges right now organizations faces is “creativity gap,” which refers to the dilemma that creative people are rarely seen at leadership position [27]. A leader looks for creative members in the team and propels them towards the leadership ladder. Again, a good leader creates more leaders [14].

Connectivity/communication/crowd control: Leaders' have an enormous power of transference, which is described as a glue [28]. Like a gel, a leader binds loosely but effectively to his team members. He leads the "crowd" synchronously towards a positive direction. He keeps the line of communication intact between different teams. He speaks clearly and without a forked tongue. In his contact, he gets genuinely personal to have bonds with his teams. And, if the situation arises, he listens with empathy and an open mind. Moreover, when his team members speak to him, he can read body language, and between the lines [29]. And, he does this with genuine respect [30]. His motto is always "we" instead of "I" [31]. He reads, focuses, reflects, and transmits that inspiration to followers [32].

Components of Solvent Aspect

Assuming the role of a follower: It is important for a leader to be visible to his team, interact regularly, and even sometimes bear the role of a follower or a ground soldier. This not only enhances his respect but also gives him more expertise in the organizational matter. Great leaders are great followers. Leadership and followership are synergists and nothing but two sides of a coin [33]. To be a good leader is said to become a good follower first [34]. Many times, in an organization, the hierarchy becomes a dynamic structure. 'It's not who you follow; it's what you follow' [35]. Leadership-followership is a different process from a manager's subordinates' contracts [36]. It is a shade of servant leadership, carrying "the characteristics of listening, empathy, healing, awareness, persuasion, conceptualization, foresight, stewardship, commitment to the growth of people, and building community" [37].

Be a catalyst: While working with team members at the ground level and becoming a part of various teams, he brings synergism to the organization. He becomes a catalyst leader. He develops, creates, inspires, and improves performances in others [38]. He becomes a catalyst for quality and performance [39]. Much like an ingredient, a good leader induces a chemical reaction. He ignites action in others, brings new ideas and effective changes. He does all without removing responsibility. A catalyst leader fosters innovation by focusing on people's potential. He collaborates and networks. He empowers, energizes, mobilizes, and aligns actions with strategy [40]. "Leading well now means finishing well later" [41]. This is an expression of shared leadership and a shade of distributive leadership [1].

Transparency: When a leader communicates more personally with his followers instead of email or other means, his transparency gets validated. As a result, problems are solved faster, team building becomes more natural, relationships grow authentically, trust gets promoted, and higher-levels of performance emerge [42]. Transparency has been described as a new superpower of leadership. It would be a fallacy to think that holding information is power. On the contrary, it prevents good ideas from coming in [43]. Transparency and honesty are a leader’s most valuable form of currency, which lies in his ability to truth-telling. Even in instances where the answer is not readily available, he errs on the side of answering truthfully, instead of buying time with half-truths or loopholes [44]. It is all about three Ts - transparency, truth, and trust [45]. 

Summary

**Figure 3:**
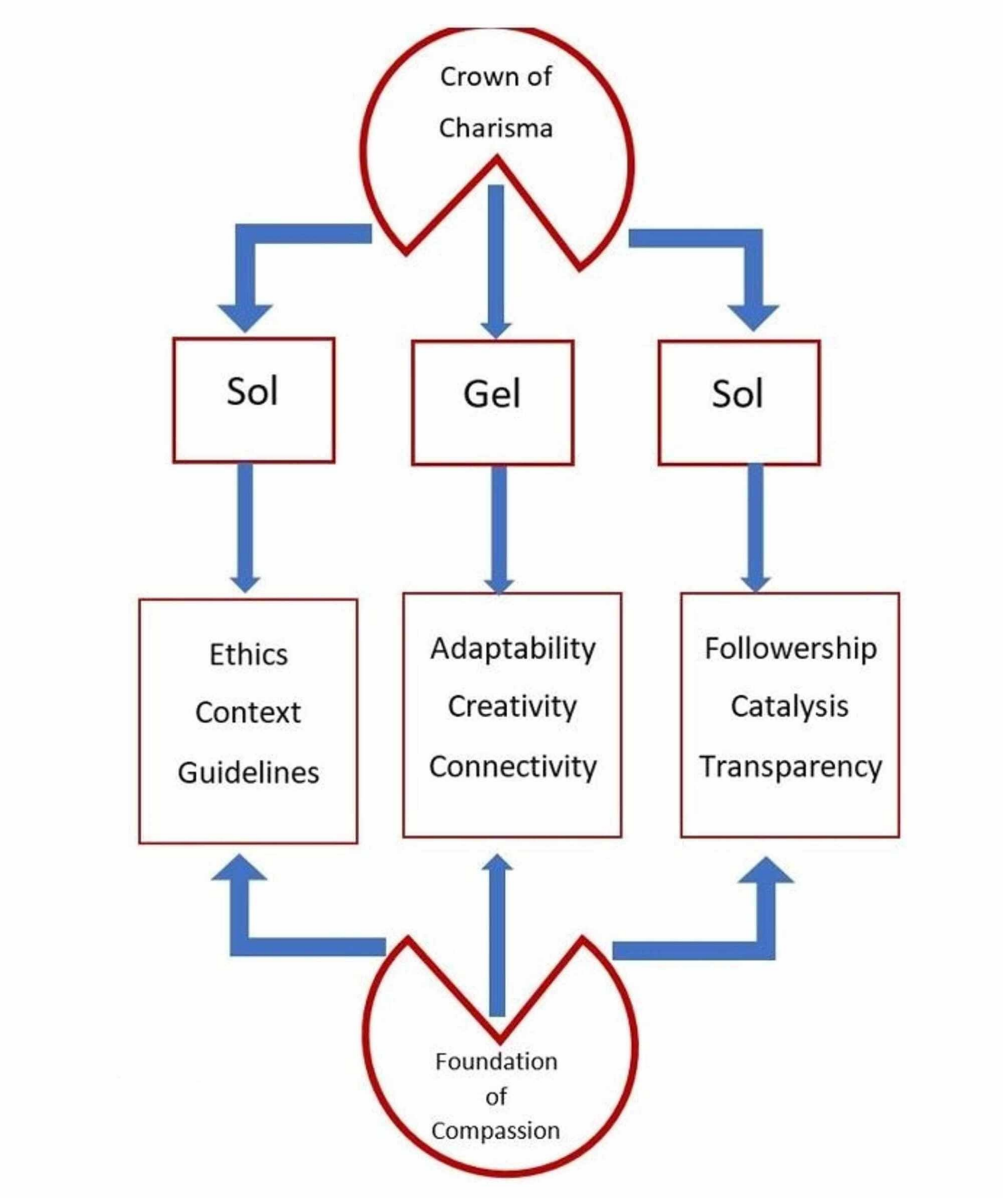
Framework of SolGelSol leadership

Charismatic leadership is an earned process. As described above in the components of SolGelSol, charisma can be achieved by integrating the above characteristics (Figure 3). It finds glitters by staying tuned, smart, and sharp to the weak signals in the organization, thereby listening, remembering, and repeating what people say using body language [46]. Confidence, creativity, vision, determination, and communication bring more jewels to this crown of charismatic leadership [47]. A charismatic leader acts with maturity and humility. It should not fall into the traps of arrogance, an inability to groom a possible successor, a power vacuum after the leader steps down, and proneness to resist addressing problems [48]. Nothing of the above can be achieved without compassion. Jinpa, the English translator for the Dalai Lama, defines compassion as “a mental state endowed with a sense of concern for the suffering of others and aspiration to see that suffering relieved.” He described three components of compassion: cognitive (“I understand you”), and effective (“I feel for you”), and motivational (“I want to help you”) [49]. The essence of compassion can be captured through empathy, sympathy, consideration, understanding, caring, concern, and the ability to collaborate [50]. And, all this cannot be more important than to an ICU leader who guides and navigates team members in probably the most stressful work in society.

## Conclusions

The concept of leadership is complex and involves an interplay of many personality traits. It is a unique skill, which is gradually learned through the integration of personal experiences with inherent supervision and organizational skills. It requires constant self-monitoring and self-improvement. The authors of this article use the term SolGelSol to highlight these characteristics. SolGelSol becomes extremely critical in the context of ICU leadership owing to the fluidity of the ICU environment. ICU offers rapidly evolving circumstances combined with the clinical complexity of cases. Therefore, good leadership skills are very crucial to the smooth functioning of an ICU.
